# Enteric parasites *Cyclospora cayetanensis* and *Cryptosporidium hominis* in domestic and wildlife animals in Ghana

**DOI:** 10.1186/s13071-024-06225-5

**Published:** 2024-05-02

**Authors:** Daniel Oduro, Esther Baafi, Philip Opoku-Agyeman, Tryphena Adams, Akweley Abena Okai, Selassie Bruku, Sandra Kyei, Phillip Banahene, Caleb Danso-Coffie, Emmanuel Boafo, Rhoda Yeboah, Godfred Futagbi, Nancy Odurowah Duah-Quashie

**Affiliations:** 1 Department of Animal Biology and Conservation Science, School of Biological Sciences, College of Basic and Applied Sciences, University of Ghana, Accra, Ghana; 2 Department of Epidemiology, Noguchi Memorial Institute for Medical Research, College of Health Sciences, University of Ghana, Accra, Ghana

**Keywords:** Enteric infections, Protozoan parasites, *Cryptosporidium sp*., *Cyclospora sp*., *Giardia sp*

## Abstract

**Background:**

Enteric parasitic infections remain a major public health problem globally. *Cryptosporidium *spp.,* Cyclospora* spp. and *Giardia* spp. are parasites that cause diarrhea in the general populations of both developed and developing countries. Information from molecular genetic studies on the speciation of these parasites and on the role of animals as vectors in disease transmission is lacking in Ghana. This study therefore investigated these diarrhea-causing parasites in humans, domestic rats and wildlife animals in Ghana using molecular tools.

**Methods:**

Fecal samples were collected from asymptomatic school children aged 9–12 years living around the Shai Hills Resource Reserve (tourist site), from wildlife (zebras, kobs, baboons, ostriches, bush rats and bush bucks) at the same site, from warthogs at the Mole National Park (tourist site) and from rats at the Madina Market (a popular vegetable market in Accra, Ghana. The 18S rRNA gene (*18S rRNA*) and 60-kDa glycoprotein gene (*gp60*) for *Cryptosporidium* spp., the glutamate dehydrogenase gene (*gdh*) for *Giardia* spp. and the* 18S rDNA* for *Cyclospora* spp. were analyzed in all samples by PCR and Sanger sequencing as markers of speciation and genetic diversity.

**Results:**

The parasite species identified in the fecal samples collected from humans and animals included the *Cryptosporidium* species* C. hominis*, *C. muris*, *C. parvum*, *C. tyzzeri*,* C. meleagridis* and *C. andersoni*; the* Cyclopora* species* C. cayetanensis*; and the* Gardia* species, *G. lamblia* and *G. muris.* For *Cryptosporidium*, the presence of the *gp60* gene confirmed the finding of *C. parvum* (41%, 35/85 samples) and *C*. *hominis* (29%, 27/85 samples) in animal samples. *Cyclospora cayetanensis* was found in animal samples for the first time in Ghana. Only one human sample (5%, 1/20) but the majority of animal samples (58%, 51/88) had all three parasite species in the samples tested.

**Conclusions:**

Based on these results of fecal sample testing for parasites, we conclude that animals and human share species of the three genera (*Cryptosporidium*,* Cyclospora*, *Giardia*), with the parasitic species mostly found in animals also found in human samples, and vice-versa. The presence of enteric parasites as mixed infections in asymptomatic humans and animal species indicates that they are reservoirs of infections. This is the first study to report the presence of *C. cayetanensis* and *C*. *hominis* in animals from Ghana. Our findings highlight the need for a detailed description of these parasites using high-throughput genetic tools to further understand these parasites and the neglected tropical diseases they cause in Ghana where such information is scanty.

**Graphical Abstract:**

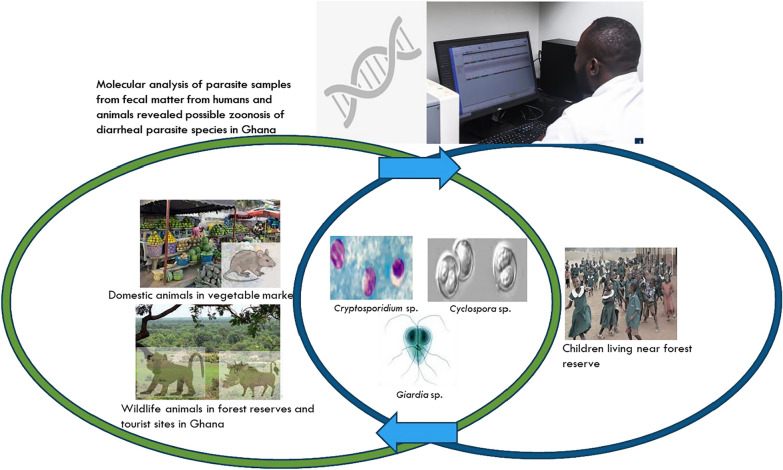

**Supplementary Information:**

The online version contains supplementary material available at 10.1186/s13071-024-06225-5.

## Background

Diarrheal diseases caused by enteric parasites remain one of the major causes of mortality worldwide [1]. An estimated 525,000 children under the age of 5 years die from diarrhea every year out of the 1.7 billion cases estimated per year [2]. Approximately 50 million people worldwide suffer from parasitic intestinal infections annually, with about 40,000–100,000 of these dying [1]. Parasites such as *Cryptosporidium* spp., *Giardia* spp. and *Cyclospora* spp. cause the diseases cryptosporidiosis, cyclosporiasis and giardiasis, respectively, and are regarded as Neglected Tropical Diseases (NTD) due to the minimal attention given to the diseases they cause [3–5]. The fecal–oral route is generally the commonest mode of transmission of these parasites. In the presence of poor sanitary conditions, infectious oocysts and cysts excreted from hosts can pollute food, water and the environment, leading to food-borne and water-borne outbreaks in humans and animals [6, 7]. Therefore, these diseases are more prevalent in developing countries of the world, although there are reports of these diseases in developed countries [8].

The WHO has identified *Cryptosporidium* sp. as the worldwide most consistent diarrhea-causing protozoan [9]. The US Center for Disease Control and Prevention (CDC) reported that about 33% of people in developing countries have experienced giardiasis [8]. This prevalence and their high socio-economic and public health burden resulted in both *Cryptosporidium* sp. and *G. duodenalis* being included in the 2004 WHO "Neglected Disease Initiative" [10]. In sub-Saharan Africa (sSA), it has been estimated that 2.9 million cases of cryptosporidiosis occur annually in children aged < 24 months [11], while a higher prevalence of giardiasis has been reported in Africa and other developing countries [12].

Food and water have been reported to be the major routes of diarrhea outbreaks in Ghana, with vegetables being a major source of contamination [13–15]. The improper storage and handling of food products, the inappropriate disposal of organic products and the widespread dispersal of garbage at the various food markets have enhanced the breeding and continuous existence of rodents which are potential carriers of the parasites. In addition, due to the euryphagic eating habits of rodents and other wildlife animals, these animals are opportunistic survivors that are often found within and near the settlements of humans, thereby enhancing the risk of zoonosis. Zoonotic diseases, mainly those pertaining to rodents and other wildlife, pose a significant threat to human health [16, 17]. Data on the prevalence of these enteric parasites in rodents and wildlife are rare although studies have been conducted on the prevalence of the parasites in cattle, rabbits, some fresh food products and humans (especially children) in Ghana [18–20]. The heavy burden of infection with these parasites and its implications in malnutrition, mortality and child growth in the country remain unknown.

Over the years, parasite detection techniques have evolved from conventional methods such as microscopy and immunologically based assays to molecular methods. Conventional methods used to identify parasite include examination of fecal smears with acid-fast stains such as Ziehl–Neelsen, which is a technique commonly used by diagnostic facilities, and microscopy. These methods are laborious and time-consuming and also require experienced microscopists to accurately identify the oocysts and cysts of these parasites. The detection limits of conventional diagnostic techniques have been reported to be as low as 50,000 to 500,000 oocysts per gram of feces for *Cryptosporidium* sp. [21] and ten to hundred cysts for *Giardia* spp. [22]. These limitations underlie the efforts to improve molecular identification techniques, such as the PCR and DNA sequencing. Studies have shown that PCR methods have a higher sensitivity and higher specificity than microscopy [23].

For *Cryptosporidium* spp., the 60-kDA glycoprotein gene (*gp60*) is the most commonly used marker for subtyping of *C. parvum* and *C. hominis* [24, 25]. Even though the *l8S rRNA* gene contains low level of intraspecific variation and is widely used, however, *gp60* contains several regions with high mutation rates, including a “hyper-variable” microsatellite region [25, 26]. Generally, for *C. parvum*, there are 19 identified *gp60* genotype families (Ila-Ilt) [25]. Families Ila and Ild are zoonotic, while families Ile and Ild are highly prevalent and widely distributed. *Cryptosporidium hominis* has 10 defined *gp60* genotype families (Ia-lk) [24, 25]. The *gdh* gene of *Giardia sp*. and the *18S rDNA* gene of *Cyclospora sp*. have also been used for speciation analysis [19, 27]. In this study, we used molecular methods to identify *Cryptosporidium* spp., *Cyclospora* spp. and *Giardia* spp., three diarrhea-causing enteric parasites, in fecal samples from humans and animals in Ghana and determine the potential for zoonotic transmission, if any, among hosts of these parasites.

## Methods

### Study design and population

The study was cross-sectional in design. The study population comprised asymptomatic school children, aged 9 to 12 years living around the Shai Hills Resource Reserve, free-ranging domestic rats from the Madina vegetable market and selected wildlife mammals from the Mole National Park and the Shai Hills Resource Reserve, respectively. The ages of the animals used in the study were not determined.

### Study sites

The study was conducted at three sites: (i) the Shai Hills Resource Reserve (5°54′N, 0°4′W); (ii) a tourist site located in the Accra plains of Ghana; and (iii) the Madina food market (5°41′0″N, 0°10′0″W), a popular vegetable market located in the La Nkwantanang–Madina Municipal District of Accra and the Mole National Park, a tourist site. The Shai-Hills Resource Reserve and the Mole National Park (covering approximately an area of 4850 km^2^ between latitudes 9°12′N and 10°12′N and longitudes 1°20′W and 2°15′W) [28] are national conservation areas south and north (savanna region) of Ghana, respectively. These sites are home to many fauna, including baboons, warthogs (mostly in Mole national Park), zebras, ostriches, bush rats and antelopes (bush bucks and kobs), with human settlements located within and outside the parks. Baboons, warthogs, and bush rats are often found in these human settlements. The Madina food market is a major food hub in Accra which serves both the local population and persons traveling to major tourist sites in the Eastern, Northern and Volta regions in Ghana. Selection of the two national parks were based on human (local and tourist)-animal interactions both within and around the parks.

### Sample collection

Fecal samples were collected from children, rats, warthogs and wild animals (kobs, baboon, bush rats, bush buck, zebra and ostriches). For human samples, fresh early morning stool samples (about 10 g) were obtained from 20 asymptomatic school of children by parents or guardians after informed consent was provided in November 2021. A total of 48 rats (*Rattus norvegicus*) were trapped individually using a tomahawk trap at different sites of the market between November 2020 and December 2020. The trapping was conducted prior to fumigation by the Madina Municipal Assembly. Samples of fecal droppings consisting of five fresh pellets (about 10 g) were collected from each trap and the rats then released. For warthogs (*Phacochoerus africanus*), with the assistance of tour guides, 10 g of freshly voided fecal samples (*n* = 20) were randomly collected early in the morning at various locations of the Staff Quarters of the Mole National Park in September 2021. The warthogs were observed from about 10 m away and the characteristic single ringform (kidney-shaped) feces of warthogs was a useful marker for ensuring that sampling the same animal twice was avoided [29]. For the other wildlife included in the study, 20 fecal samples (10 g) each were collected from kobs (*Kobus kobs*, *n* = 10), baboon (*Papio Anubis*, *n* = 1), bush rats (*Thryonomys swinderianus*, *n* = 3), bush buck (*Tragelaphus scriptus*, *n* = 2), zebra (*Equus quagga*, *n* = 2) and ostriches (*Struthio camelus*, *n* = 2) from the Shai Hills Resource Reserve. Fecal samples from kobs were collected 20 m apart at their grazing sites while those of bush bucks were collected at three different hideouts on the same morning inside the Shai hills resource reserve. The zebras and ostriches were confined in separate enclosures within the Shai hills. Bush rats were trapped and released after the collection of fresh fecal droppings. A fecal sample from the baboon was collected at a Staff residence within the Shai hills after observing the animal from about 10 m away. All fecal/stool samples were collected into sterile sample vials and stored at − 20 °C for 2 months. None of the samples were watery diarrheal stools.

The animals were selected based on their close proximity to humans: the rats come into contact with humans daily at the market; the warthogs found at the Mole National Park live within the human settlement and in the park, interacting with both the locals and tourists; and zebras, baboons, kobs, ostriches and bush bucks from the Shai Hills Resource Reserve interact daily with the visitors and with the park guides. The required sample size for humans and animals was determined based on two independent study groups [30] and using previous reports on the prevalence of protozoan parasites in wild animals (42%) and human (8.4%) [20].

### Molecular detection of parasite genera and species

Total DNA was extracted from all fecal/stool samples using the QIAamp Fast DNA Stool Mini Kit following the manufacturer's protocol (QIAGEN, Hilden, Germany). The extracted DNA was quantified using the Nanodrop spectrophotometer (Thermo Fisher Scientific, Waltham, MA, USA) to determine the concentration and purity of the extracted DNA. The purified DNA were used for the nested PCRs for: the *18S rRNA* gene of *Cryptosporidium* spp., the *gdh* of *Giardia* spp. and the *18S rDNA* gene of *Cyclospora* spp. following published protocols [18, 20, 27]. All primers and cycling conditions used for the PCRs are shown in Additional file 1: Table S1. Each PCR was performed in a total volume of 30 µ1 consisting of 1× Kapa Master Mix, sterile double distilled water, l0 µM of each primer and 3 µl of the extracted DNA. A positive control from a previous study and a negative control of no template were also used in the PCR analyses. All PCR products were resolved in a 2% agarose gel and visualized with SYBR Safe DNA Gel Stain (Thermo Fisher Scientific). Amplicons were sequenced at Macrogen Europe BV (Amsterdam, The Netherlands). To further distinguish and ascertain the presence of *C. parvum* and *C. hominis* in samples, subgenotyping of *gp60* was performed using nested PCR following published protocols [31]. Briefly, the PCR was performed in a total volume of 30 µl consisting of 1× Go Taq Green Master Mix (Promega, Madison, WI, USA), sterile double distilled water, l0 µM of each primer and 5 µl of the extracted DNA. The *gp60* PCR amplicons were also sequenced.

### Data analysis

Consensus sequence editing was carried out using Benchling.com (San Francisco, CA, USA). Sequences with low-quality scores (< 40% coverage) were not included in the analysis. Quality sequences obtained were run in the Basic Local Alignment Tool (BLAST) (http://blast.ncbi.nlm.nih.gov/) to check for authenticity of the sequence data and for species identification. Identification of the species was done by blasting and comparing both forward and reverse sequences with those from GenBank using the NCBI nucleotide BLAST (http://blast.ncbi.nlm.nih.gov/) to detect regions of local similarity. Other gene databases used for comparison were Crypto DB (https://cryptodb.org/cryptodb/app/workspace/blast/new) to identify *Cryptosporidium* spp., Toxo DB (https://toxodb.org/toxo/app/workspace/blast/new) for *Cyclospora* spp. and *Giardia* DB (https://giardiadb.org/giardiadb/app/workspace/blast/new) for *Giardia* spp. Database sequences similar to sequences obtained from the study were based on the expected value (E), maximal identity and score, query coverage and total score. The proportion of individuals with the protozoan parasites were determined using Microsoft Excel (Microsoft Corp., Redmond, WA, USA). Using GraphPad Prism version 10.2.0 (GraphPad Software, San Diego, CA, USA), we performed Kruskal-Wallis tests to compare proportions of parasites in humans and other animal groups.

## Results

### Prevalence of enteric protozoan parasites

The PCR amplification and DNA sequencing procedures were successful for all genes of the parasite genera under study for 108 samples (20 humans, 48 domestic rats, 20 warthogs, 20 other wildlife animals [2 zebras, 1 baboon, 2 bush bucks, 3 bush rats, 2 ostriches, 10 kobs]). The genetic analysis revealed that of the 20 human samples analyzed, 70% (14/20), 10% (2/20) and 75% (15/20) harbored *Cryptosporidium* spp., *Cyclospora* spp. and *Giardia *spp., respectively. For the animal samples, of the total of 88 samples analyzed, *Cryptosporidium* sp. was identified in 97% (85/88) of samples; *Cyclospora* sp. was identified in 88.6% (78/88) of samples; and *Giardia* sp. was identified in 78.4% (69/88) of samples. Table 1 shows the proportions of the three protozoan parasites in the various groups assessed. There was a significant difference between the proportions of *Cryptosporidium* spp. and *Cyclospora* spp. in the various groups (*P* = 0.001). The highest proportions of *Cryptosporidium* spp. (93.8%) and *Cyclospora* spp. (95%) were recorded in domestic rats and other wild animals, respectively, with the lowest proportions of these two protozoan parasites recorded in humans (Table 1)**.** The proportion of *Giardia* spp. was not significantly different (*P* = 0.781) in the groups assessed even though humans were observed to have the highest percentage (75%)**.**

**Table 1:** Proportions of *Cryptosporidium* sp., *Cyclospora* sp. and *Giardia* sp. in fecal samples collected from humans and animals based molecular analysis of *18S rRNA*, *18S rDNA* and *gdh* , respectively

Groups	Number of fecal samples	*Cryptosporidium*			*Cyclospora*			*Giardia*		
		Number of positive samples (%)	95% CI	*P*-value	Number of positive samples (%)	95% CI	*P*-value	Number of positive samples (%)	95% CI	*P*-value
Human	20	14 (70)	48.10–85.45	0.001	2 (10)	1.77–30.10	< 0.001	15 (75)	53.13–88.81	0.781
Domestic rats	48	45 (93.8)	63.96–94.76		42 (93.33)	82.14–97.71		33 (68.75)	54.67–80.05	
Warthogs	20	20 (100)	83.89–100		17 (85)	63.96–94.76		12 (60.00)	38.66–78.12	
Other wildlife	20	20 (100)	83.89–100		19 (95)	76.39–99.74		14 (70)	48.10–85.45	
*Baboon*	1	1 (100)	5.13–100		1 (100)	5.13–100		1 (100)	5.13–100	
*Zebra*	2	2 (100)	17.77–100		2 (100)	17.77–100		2 (100)	17.77–100	
*Bush rats*	3	3 (100)	43.85–100		2 (66.7)	11.85–98.29		3 (100)	43.85–100	
*Bush buck*	2	2 (100)	17.77–100		2 (100)	17.77–100		0 (100)	0.00–82.23	
*Ostrich*	2	2 (100)	17.77–100		2 (100)	17.77–100		2 (100)	17.77–100	
*Kob*	10	10 (100)	72.25–100		10 (100)	72.25–100		6 (60)	31.27–83.18	

*CI* Confidence interval

### Proportions of parasites species and strains in humans and animals

Molecular identification of *Cryptosporidium* spp. using both *18S rRNA* and *gp60* showed revealed presence of six *Cryptosporidium* species: *C. parvum*, *C. hominis*, *C. meleagridis*, *C. muris*, *C. tyzerri* and *C. andersoni* (Table 2). *Cryptosporidium parvum* (Iowa II) was observed in 70% of the human samples while *C. hominis* was more prevalent in animals. This is the first study to observe *C. hominis* in animals from Ghana. All other *Cryptosporidium* spp. (except for *C. parvum and C. hominis)* were prevalent in the animal samples only (Table 2). The only *Cryptosporidium* spp. identified in the human samples were *C. parvum* and *C. hominis*.

**Table 2:** Proportions of species of the three enteric protozoan parasites in the human and animal samples

Protozoan parasites	Species proportions (%)				
	Humans	Domestic rats	Warthogs	Other wildlife	*P*-value
*Cryptosporidium* spp.					
* C. hominis*	30	62.5	70	85	0.006
* C. parvum* (Iowa II)	70	45.8	40	35	
* C. muris (*RN66)	0	33.3	25	5	
* C. meleagridis* (UKMEL1)	0	10.4	15	0	
* C. tyzzeri* (UGA55)	0	2.1	10	0	
* C. andersoni* (30847)	0	2.1	5	0	
*Cyclospora* sp.					
* C. cayetanensis* (NFI_C8)	10	29.2	85	90	0.02
* C. cayetanensis* (CHN_HENO1)	0	2.1	0	5.0	
*Giardia* spp.					
*G. muris* (Roberts-Thomson)	0	14.6	10	25	0.03
*G. lamblia*	75	54.2	50	45	
* G. lamblia* (Assemblage A2 isolate DH)	0	20.8	10	25	0.77
* G. lamblia* (Assemblage A isolate WB)	75	0.0	5	0	
* G. lamblia* (Assemblage B isolate GS)	0	18.8	20	15	
* G. lamblia* (Assemblage E isolate P1S)	0	14.6	15	5	

Two strains of *C. cayetanensis* were identified, NFI_C8 and the Chinese strain CHN_HENO1. Of these two stains, *C. cayetanensis* NF1_C8 was the most prevalent, identified in 72% (78/108) of all samples. *Cyclospora cayetanensis* strain NF1_C8 was found in the highest proportion in warthogs and the other wildlife in each group (90%, 18/20), followed by rats (85%, 41/48). This is the first report of *Cyclospora sp.* in animals from Ghana. The *C. cayetanensis* CHN_HENO1 strain was identified in two animal samples only, and the *C. cayetanensis* NF1_C8 strains was found only in two human samples (10%, 2/20).

The analysis for *Giardia* spp. revealed only two species in all samples: *G. lamblia (*56%, 60/108) and *G. muris* (13%, 14/108). Five different strains of *G. lamblia* were observed: *G. lamblia* Assemblage E P15 (18.3%, 11/60), *G. lamblia Assemblage* B isolate GS_B (5.0%, 3/60), *G. lamblia* Assemblage A2 isolate DH (28.3%, 17/60), *G. lamblia* Assemblage B isolate GS (21.7%, 13/60) and *G. lamblia* Assemblage A isolate WB (26.7%, 16/60). *Gardia muris* was observed only in the animal samples, and *G. lamblia* was more prevalent in human samples than in animal samples (*P* = 0.03). Other strains showed varied proportions of presence in the animal samples (Table 2). Only *G. lamblia* Assemblage A isolate WB was identified in human sample, with as high as 75% (15/20) of the participants infected with this parasite type; interestingly only warthogs shared this strain with humans.

### Polyparasitism in humans and animals

**Fig. 1:**
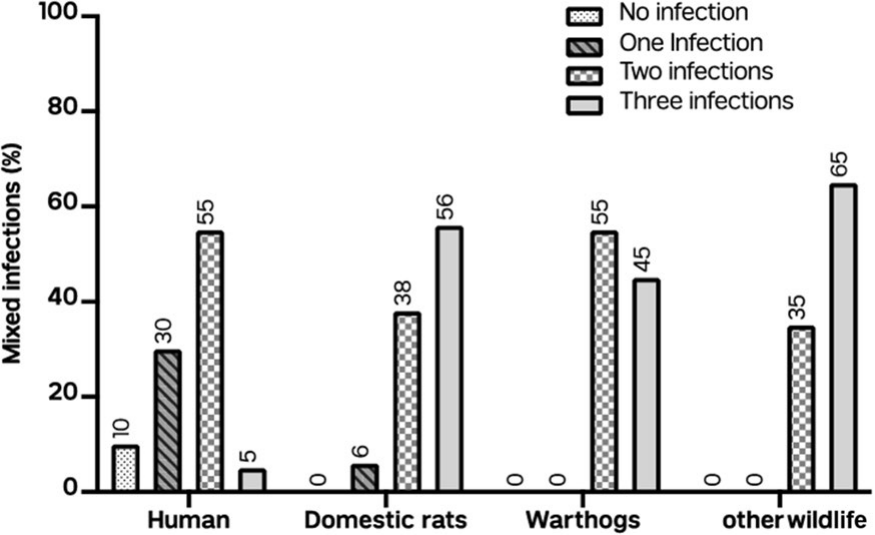
Concurrent enteric infections in the human and selected animal samples. Most animals studied carried all three protozoan parasite species

**Fig. 2:**
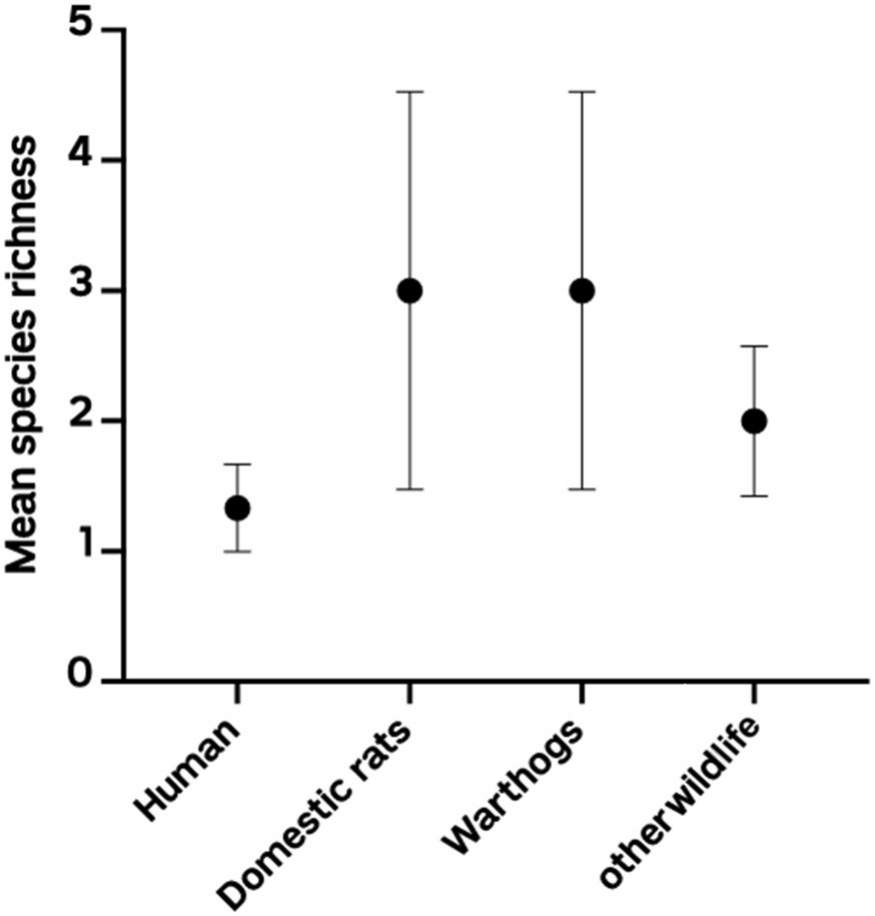
Enteric species richness in human and animal hosts. The highest number of parasite species were recorded in the domestic rat and warthog populations. Fewer species of the three protozoan parasites were recorded in humans

All fecal samples analyzed for both humans and animals revealed coinfections of all parasites in these carriers of infection. Only one human had a coinfection with three parasites (5%, 1/20), with the majority having two parasites (55%, 11/20). Most of the animals were coinfected with three parasites (58%, 51/88). Based on the fecal samples, 45% (9/20) and 55% (11/20) of the warthogs were coinfected with two and three parasites, respectively. Most of the coinfections in the rats were three parasites (60%, 29/48), followed by two parasites (27%, 13/48). A similar observation was made in the other wildlife samples, with the majority of animals having three parasites (65%, 13/20) and a minority having two parasites (35%, 7/20). The distribution of coinfections in humans and animals and the species richness are shown in Figs. 1 and Fig. 2, respectively.

## Discussion

The impact of enteric parasitic infections on human health and global development is enormous as these infections affect both the general populations of both developed and developing countries [2]. These parasitic infections are considered to be neglected due to the persistent high incidence of bacterial and viral enteric infections. In Ghana, information on these enteric protozoan parasites is scanty, especially on their genetic diversity and transmission dynamics.

The choice of the animals to be assessed in this study was based on proximity to human settlements and possible interactions with local inhabitants and tourists in their habitats. Our findings portray possible human-animal interactions and consequent zoonosis with the observed shared parasite species. This study provides preliminary data on enteric protozoan parasites, specifically *Cryptosporidium* spp., *Giardia* spp. and *Cyclospora* spp., in domestic rats from a popular food market (Madina Market) in Accra that attracts both locals and visitors to the city, warthogs from the Mole National Park (a highly sought-out tourist site) and other wildlife animals from the Shai Hills Resource Reserve. The findings show the presence of *C. cayetanensis* and *C. hominis* in animals from Ghana for the first time.

The observation of *Cryptosporidium* spp. in majority of the human and animal fecal samples analyzed is intriguing. All of the humans and animals from whom samples were collected were assessed being asymptomatic for all three parasites, suggesting that some human and animal populations may serve as reservoirs of these diarrheal infections. Of the 19 *Cryptosporidium* spp. identified to date,* C. hominis*,* C. parvum*,* C. meleagridis*,* C. canis* and *C. felis* are known to be prevalent in humans [32]. Analysis of the sequenced data from the current study showed the presence of mostly *C. parvum* and *C. hominis* in human samples, with proportions of 70% and 30%, respectively. These two species are known to be responsible for about 95% of human infections, with *Cryptosporidium* spp. *C. meleagridis*,* C. canis*,* C. ubiquitum* and *C. felis* accounting for the remaining infections [33]. Unlike humans, analysis of the animal samples showed that most of the animals had mixed infections of common animal and human *Cryptosporidium* spp. that included *C. muris*,* C. meleagridis*,* C. andersoni* and *C. tyzzeri*, all of which have also been reported in other human studies [34, 35].

Humans are the major hosts for *C. parvum* and *C. hominis*; however, there have been several reports of these species in wild animal hosts and non-human primates, thus increasing the probability of zoonotic transmission [36, 37]. In our study, we observed both *C. parvum* and *C. hominis* in animal fecal samples, with the latter present at the higher proportion (> 60% in some samples). A number of studies have reported *C. hominis* in horses and other animals, while *C. parvum* has been recorded in ruminants [20, 38–41]. In the present study *C. hominis* was more prevalent (> 60%) in the animal samples. To date, around nine species of *Cryptosporidium* have been identified in rats, with the majority being *C. parvum* and* C. muris*, but not *C. hominis* [42]. *Cryptosporidium muris* was not detected in the human fecal samples in the current study; however, it is worth mentioning this parasite species and *C. ubiquitum* have been considered emerging zoonotic species as they have been detected in humans and wildlife [43, 44].

The presence of *C. cayetanensis* (NFI_C8) in samples of humans and wild animal groups in this study represents a significant finding because such an investigation has been uncharted and therefore not much work has been done in Ghana. The proportion of *C. cayetanensis* in humans was quite low (10%) in this study but high in wild animals (> 80%). Previous studies that investigated *Cyclospora spp*. in humans and animals (chimpanzees, macaques, dogs, chicken, and monkeys) showed a proportion range of 5–47.7% [45–47]. Interestingly, *C. cayetanensis* is known to be the only species that is infective to humans, therefore the high proportion of this isolate observed in animals in this study is quite disturbing. Data on *Cyclospora spp*. in rodents and wildlife is scarce, thus the zoonotic potential of this enteric parasite remains unknown. Results of this current study could imply the potential of zoonotic transmission of this enteric parasite in Ghana.

*Giardia lamblia* (also known as *G. intestinalis* or* G. duodenalis*) was the most prevalent of the parasites tested in both human (75%) and animal (> 40%) samples. These findings corroborate those of previous studies which reported the presence of *Giardia* spp. in a wide range of animal hosts and humans globally [39, 48–51]. There are currently seven assemblages of *G. lamblia*, designated with the letter A to G. of which three (A, B and E) were identified in this study. Assemblage A consists of mostly two subgroups, Al and A2, and both Assemblages A and B have been reported to infect humans and other mammals such as livestock, cats, wild animals and dogs [52, 53]. Assemblage A was the most prevalent in the human samples and was also seen in a warthog. Although the warthog samples and that of the human participants were obtained from different locations, the presence of Assemblage A isolate WB in both humans and warthog should be of concern. It is worth noting that *G. lamblia* Assemblages A and B are known to have zoonotic potential because both are the only genotypes observed in both humans and animals [54].

We considered that the animals and study sites selected for the present study were appropriate within the framework of our study aim, which was to identify the effect of the close association between humans and animals, whether domestic, wildlife or tourist sites. The work reported in this study is the most extensive genetic analysis of these three enteric parasitic infections conducted in Ghana so far, and the potential of zoonotic transmission of these parasites is very clear due to the shared species by both humans and animals. The genetic analysis conducted to identify the parasites to the species level provides data which helps ascertain the enteric protozoan parasites being circulated in Ghana in both humans and animals and confirms the possibility of zoonosis.

## Conclusions

The findings of this study highlight the presence of at least one of the enteric parasites *Cryptosporidium* spp., *Cyclospora* spp. and *Giardia* spp. in all animals and about 90% of children assessed. The study results affirmed the presence of these enteric parasites in fecal samples. Noting that some of the parasite species identified have zoonotic potential, there is a great risk for cryptosporidiosis, cyclosporiasis and giardiasis transmission in Ghana. This is the first study to report *C. cayetanensis* and *C. hominis* in animals from Ghana and supports the need for using high-throughput genetic tools to improve our understanding these neglected tropical diseases in Ghana where there is limited information.

### Supplementary Information

**Additional file 1: Table S1.** Amplified genes, primer sequences, sizes of PCR amplicons and cycling conditions for the molecular identification of *Cryptosporidium* spp., *Cyclospora* spp. and *Giardia* spp.

## Data Availability

All data have been described in the body text. The sequences of the parasites have been deposited at the NCBI Genbank under accession numbers OQ995305-OQ995315 and OR371752-OR371762.

## Abbreviations

CDC: Center for Diseases Control and Prevention
*gdh*: Glutamate dehydrogenase gene
*gp60*: 60-kDa glycoprotein gene
PCR: Polymerase chain reaction
WHO: World Health Organization
NTD: Neglected Tropical Diseases
rDNA: ribosomal Deoxyribonucleic acid
rRNA: ribosomal Ribonucleic acid
sSA: Sub-Saharan Africa
