# Supplementary material for: Enteric parasites Cyclospora cayetanensis and Cryptosporidium hominis in domestic and wildlife animals in Ghana
Source: Parasit Vectors. 2024 May 2;17:199. doi: 10.1186/s13071-024-06225-5 (PMC11064306; doi:10.1186/s13071-024-06225-5)
Supplement: Supplementary file 1 — Additional file 1: Table S1. Amplified genes, primer sequences, sizes of PCR amplicons and cycling conditions for the molecular identification of Cryptosporidium spp., Cyclospora spp. and Giardia spp. [file 13071_2024_6225_MOESM1_ESM.docx]

**Additional file 1: Table S1.** **Genes amplified, primer sequences, sizes of PCR amplicons and cycling conditions for the molecular identification of** ***Cryptosporidium spp., Cyclospora spp*. and *Giardia spp*.**

| **Parasite** | **Gene amplified** | **Primer sequence** | **PCR product size/bp** | **Cycling conditions** |
| --- | --- | --- | --- | --- |
| *Cryptosporidium spp.* | *18sRNA* | Primary amplification  Forward- NDIAGF2  5'- CAATTGGAGGGCAAGTCTGGTGCCAGC-3'  Reverse-NDIAGR2  5'- CCTTCCTATGTCTGGACCTGGTGAGT-3'  Secondary amplification  Forward-DIAGF  5'- AAGCTCGTAGTTGGATTTCTG-3'  Reverse-DIAGR  5'- TAAGGTGCTGAAGGAGTAAGG-3' | 655 – 667  435 | 94^o^C - 3 mins;  94^o^C - 45 s, 58^o^C - 45 s, 72^o^C - 1 min (40X);  72^o^C - 7 mins  94^o^C - 3 mins;  94^o^C - 45 s, 55^o^C - 45 s, 72^o^C - 1 min  (45X);  72^o^C - 7 mins |
|  | *gp60* | Primary amplification  Forward - AL3531  5’-ATAGTCTCCGCTGTATTC-3’  Reverse - AL3534  5’-GCAGAGGAACCAGCATC-3’  Secondary amplification  Forward - AL3532  5’-TCCGCTGTATTCTCAGCC-3’  Reverse -AL3533  5’-GAGATATATCTTGGTGCG-3’ | 800-850 | 94°C - 3 mins;  94°C - 45 s, 54.1°C - 45 s, 72°C - 1 min (30X);  72°C - 10 mins. |
| *Giardia spp* | *gdh* | Primary amplification  Forward-GDH1a  5'- ATCTTCGAGAAGGATGCTTGAG-3'  Reverse-GDH5s  5'- GGATACTTSTCCTTGAACTC-3'  Secondary amplification  Forward-GDHeF  5'- TACACGTYAAYCGYGGYTTCCGT-3'  Reverse-GDHiR  5'- GTTRTCCTTGCACATCTCC-3' | 2324  461 | 94^o^C - 7 mins;  94^o^C - 1 min, 56^o^C - 1 min, 72^o^C - 1 min (35X);  72^o^C - 7 mins  94^o^C - 30 s, 56^o^C - 20 s, 72^o^C - 45 s (56X);  72^o^C - 7 mins |
| *Cyclospora spp.* | *18sDNA* | Primary amplification  Forward-ExCycF  5'- AATGTAAAACCCTTCCAGAGTAAC-3'  Reverse-ExCycR  5'- GCAATAATCTATCCCCATCACG-3'  Secondary amplification  Forward-NesCycF  5'- AATTCCAGCTCCAATAGTGTAT-3'  Reverse-NesCycR  5'- CAGGAGAAGCCAAGGTAGGCRTTT-3' | 1747bp  500bp | 94^o^C - 7 mins;  94^o^C - 1 min, 55^o^C - 1 min, 72^o^C - 1 min (35X);  72^o^C - 7 mins  94^o^C - 7 mins;  94^o^C - 1 min, 55^o^C - 1 min, 72^o^C - 1 min (35X);  72^o^C - 7 mins |
